# Melanoma disparities in Utah: Disproportionate rates among Hispanic and non-White populations in a population-based analysis

**DOI:** 10.1016/j.jdin.2026.03.016

**Published:** 2026-05-12

**Authors:** David Hoyt, Austen Callister, Kimberly Herget, Jonas A. Adalsteinsson, Douglas Grossman, Sancy Leachman, Alice Frigerio

**Affiliations:** a University of Utah School of Medicine, Salt Lake City, Utah; b Utah Cancer Registry, Salt Lake City, Utah; c Faculty of Medicine, University of Iceland, Reykjavik, Iceland; d Department of Dermatology, Landspitali National-University Hospital, Reykjavik, Iceland; e Department of Dermatology, University of Utah Health Sciences Center, Salt Lake City, Utah; f Huntsman Cancer Institute, Salt Lake City, Utah

**Keywords:** epidemiology, incidence, melanoma, mortality, oncology, SEER database, skin cancer

*To the Editor:* Using Surveillance, Epidemiology, and End Results (SEER) data from 2000-2021, we compared melanoma incidence in Utah with pooled remaining 16 registries in the SEER 17 database (non-Utah SEER 17 registries), which covers 26.5% of the U.S. population. Age-adjusted rates per 100,000 person-years were analyzed using Joinpoint regression and annual percent change (APC). Because the SEER database is publicly available, this study is exempt from institutional review board oversight.

We identified striking racial and ethnic disparities in melanoma incidence in Utah. Although melanoma incidence was elevated across groups in Utah, including non-Hispanic White populations, the magnitude of elevation was disproportionately greater in several minority populations. Lentigo maligna melanoma (LMM) incidence was 8.7 times higher in Hispanic individuals and 14.2 times higher in non-White populations compared with their counterparts in non-Utah SEER 17 registries. Asian and Pacific Islanders experienced a threefold higher total melanoma incidence in Utah than non-Utah SEER 17 registries. These data reveal a pronounced melanoma burden among Utah’s minority groups.

Overall melanoma incidence in Utah was expectedly elevated compared with national averages.^1^ While nodular melanoma predominates nationally, we found that Utah’s LMM rate was 4 times higher than its nodular melanoma rate. *In situ* lesions (including melanoma in situ (MIS) and lentigo maligna (LM)) rose exponentially in Utah (APC 5.26%, *P* < .001), surpassing invasive melanoma incidence in 2019 and continuing upward (Fig 1). This change reflects growing predominance of early-stage lesions in Utah.

**Fig 1:**
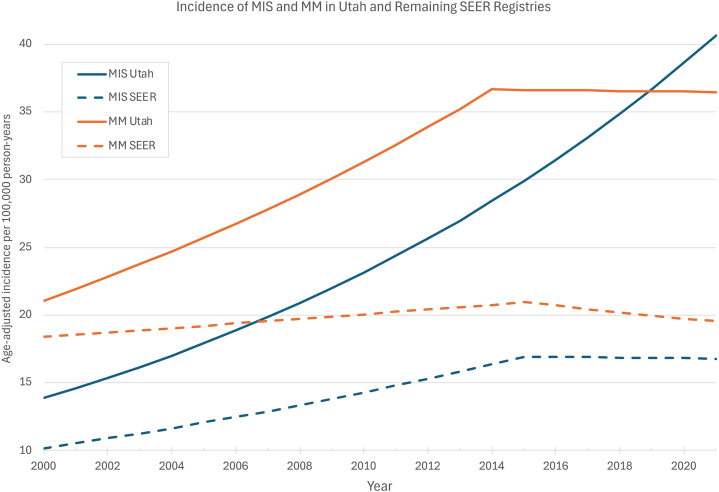
Melanoma in situ and malignant melanoma age-adjusted incidence in Utah and remaining SEER registries, 2000-2021.

Age-specific trends elucidate demographic shifts. Adults aged 75 years and above had the highest melanoma incidence, with the fastest increase occurring among elderly women (APC 4.28%, *P* < .001). Utahns <50 years had a nearly two-fold increase in total melanoma, primarily driven by MIS. Despite increased incidence, melanoma mortality declined in all regions over the study period, which may be attributed to earlier detection and improved therapies.

While overdiagnosis may contribute to increasing melanoma incidence,^2^ the unique disparities in Utah’s melanoma trends suggest other factors may be at play. Utah’s high UV exposure and outdoor culture are well-established risk factors, likely contributing to elevated melanoma rates. However, the markedly high rates of LMM among Hispanic and non-White populations suggest additional factors may contribute. We hypothesize that skin cancer prevention and awareness efforts may historically have been directed toward populations perceived as higher risk, potentially leaving minority populations without targeted prevention messaging despite shared high UV exposure. Differences in access to dermatologic care may also contribute to diagnostic patterns that differ from national trends.^3^

These findings suggest a need to expand skin cancer education and prevention strategies beyond traditionally high-risk White populations in Utah. Melanoma incidence was elevated across groups in Utah, but the relative increase was disproportionately greater in several minority populations, underscoring the need for more inclusive prevention and screening efforts. Substantial increases in LM and MIS may indicate the need for further diagnostic guidelines to maximize early detection while mitigating harms from overdiagnosis.^4^^,^^5^ Utah’s melanoma profile exemplifies the need for monitoring and adaptation of education and prevention, particularly in regions with high UV exposure and shifting demographics (Table I).

**Table I:** Melanoma subtype age-standardized incidence by ethnoracial group in Utah and remaining SEER registries, 2000-2021

Subtype	Race/ethnicity	Utah			Remaining SEER			Ratio†	*P*-value
		Rate∗	95% CI	*N*	Rate∗	95% CI	*N*		
Melanoma In Situ	All	13.87	13.6-14.2	7170	11.07	11.0-11.1	204,103	1.25	<.0001
	Non-Hispanic white	15.08	14.7-15.5	6766	15.00	14.93-15.07	177,365	1.01	.6584
	Hispanic	3.59	2.98-4.28	155	2.00	1.94-2.06	5257	1.80	<.0001
	Non-Hispanic, non-white	1.34	0.89-1.94	31	0.456	0.43-0.48	1683	2.94	<.0001
Lentigo maligna	All	12.42	12.1-12.7	6078	3.49	3.47-3.52	64,129	3.55	<.0001
	Non-Hispanic White	13.23	12.9-13.6	5831	4.67	4.63-4.71	58,594	2.83	<.0001
	Hispanic	2.92	2.26-3.69	82	0.53	0.50-0.57	1190	5.47	<.0001
	Non-Hispanic, non-white	1.20	0.73-1.83	23	0.10	0.089-0.11	343	12.0	<.0001
Superficial spreading	All	13.89	13.6-14.2	7274	6.07	6.03-6.11	111,096	2.29	<.0001
	Non-Hispanic white	15.37	15.0-15.7	6925	8.89	8.83-8.94	100,459	1.73	<.0001
	Hispanic	2.81	2.33-3.36	148	1.11	1.07-1.15	3235	2.53	<.0001
	Non-Hispanic, non-white	1.16	0.79-1.66	33	0.244	0.23-0.26	914	4.77	<.0001
Lentigo maligna melanoma	All	6.12	5.9-6.4	2975	1.23	1.21-1.25	22,495	4.98	<.0001
	Non-Hispanic White	6.46	6.22-6.70	2836	1.67	1.65-1.69	20,947	3.86	<.0001
	Hispanic	1.50	1.05-2.06	43	0.172	0.15-0.19	386	8.74	<.0001
	Non-Hispanic, non-white	0.57	0.27-1.02	11	0.04	0.03-0.05	137	14.2	<.0001
Nodular	All	2.23	2.1-2.4	1116	1.41	1.39-1.43	25,776	1.58	<.0001
	Non-Hispanic white	2.44	2.3-2.6	1081	1.99	1.96-2.02	23,804	1.23	<.0001
	Hispanic	0.66	0.39-1.05	23	0.48	0.45-0.50	1193	1.40	.2239
Acral lentiginous	All	0.39	0.34-0.45	193	0.20	0.19-0.21	3628	1.97	<.0001
	Non-Hispanic White	0.41	0.35-0.47	177	0.20	0.19-0.21	2356	2.04	<.0001
	Hispanic	0.34	0.16-0.62	12	0.27	0.25-0.29	643	1.26	.5699
Desmoplastic	All	0.36	0.31-0.42	172	0.22	0.21-0.23	4057	1.60	<.0001
	Non-Hispanic white	0.37	0.32-0.44	163	0.31	0.30-0.32	3808	1.20	.0274
Amelanotic	All	0.070	0.05-0.10	33	0.081	0.077-0.086	1492	0.86	.4492
	Non-Hispanic White	0.08	0.05-0.11	32	0.12	0.111-0.124	1418	0.64	.0091

Data excluded for cohorts with ≤10 cases.

∗ Rates reported per 100,000 person-years.

† Ratios represent Utah rates divided by remaining SEER rates.

### Declaration of generative AI and AI-assisted technologies in the writing process

During the preparation of this work the author(s) used OpenEvidence in order to aid in the literature review. After using this tool/service, the author(s) reviewed and edited the content as needed and take(s) full responsibility for the content of the publication.

## Conflicts of interest

Hoyt, Callister, Herget, Adalsteinsson, Grossman, Leachman, and Frigerio report no conflicts of interest or competing interests. They alone are responsible for the content and writing of the paper; no external entity influenced the analysis or conclusions.
